# From local to global – Contributions of Indian psychiatry to international psychiatry

**DOI:** 10.4103/0019-5545.69202

**Published:** 2010-01

**Authors:** R. Srinivasa Murthy

**Affiliations:** Association for the Mentally Challenged, Dharmaram College P.O, Bangalore – 560 029, India

**Keywords:** Indian Psychiatry, global psychiatry, classification, community care, family, religion, yoga, psychotherapy

## Abstract

Indian psychiatrists have actively engaged with world psychiatry by contributing to understanding and care of persons with mental disorders based on the religious, cultural and social aspects of Indian life. The contributions are significant in the areas of outlining the scope of mental health, classification of mental disorders, understanding the course of mental disorders, psychotherapy, traditional methods of care, role of family in mental health care and care of the mentally ill in the community settings.

## INTRODUCTION

During the last one year, I have read four books of importance on the topic under consideration in this article. These books are: The Joy of Mental Health by Prof. N.N.Wig, in which he presents his understanding of mental health in the Indian context as well as interprets some of the Indian concepts like Vanaprastha and its applicability in the modern world, for the wider world audience.[1] The second book by Prof. Kapur covers the larger canvas of the ascetism in the Indian seers and his personal experiences of yoga, and reflects on the wider importance of Indian spirituality and mental health.[2] The third book is by Prof. Ajita Chakraborty. In this book of memoirs and essays Prof. Chakraborty presents the Indian view of transcultural psychiatry and how it differs from what is considered transcultural psychiatry by the western professionals. She makes a strong point by stating that ‘an Indian school of psychiatry must develop to suit Indians who are not to be seen just as a variant of westerners’.[3] The fourth book is a compilation of articles covering the pluralistic therapies and concepts of India, and their place in the larger field of mental healthcare.[4] I would like to use these as representative of the way Indian psychiatry has influenced global psychiatry. By the very nature of the subject, as well as the limitations of space, I cannot be comprehensive in the coverage and restricted to illustrate the aspects I consider as significant.

There are two aspects of Indian Psychiatry that are worth recalling. First, India has a tradition of understanding mental disorders as part of Ayurveda, as well as having a number of treatment approaches (pharmacological, psychosocial, and spiritual) for the care of mentally ill persons. This knowledge pool has continuously interacted with the international developments and enriched international psychiatry. Second, in the field of understanding of mental disorders and care of the persons with mental disorders, Indian psychiatrists have contributed to world psychiatry.[5] It is important to keep in mind this dual nature of Indian contributions when addressing this topic. In covering the Indian Psychiatric Research Contributions to International Psychiatry, it is also important to recognize that this has been a two-way process. The seven areas chosen are the following [Table 1]

**Table 1:** Indian psychiatric research contributions to international psychiatry

Scope of mental health
Classification of mental disorders
Family involvement in mental healthcare
Psychotherapy
Understanding of the mental disorders
Community care of the mentally ill persons
Traditional systems of mental healthcare

### Scope of mental health

A striking aspect of Indian psychiatry has been the broader approach to mental health, in contrast to the focus of mental disorders in most of the other countries. Indian religions and culture have placed a high value on mental health. There has been an emphasis on the prevention and promotion of mental health, along with the care of the persons with mental disorders.

Dr. Govindaswamy[6] outlined this, in the early 1950s, as follows:

“The field of mental health in India has THREE objectives. The first of these has to do with mentally ill persons; for them the objective is the restoration of health. A second has to do with those people who are mentally healthy, but who may become ill if they are not protected (prevention) from conditions that are conducive to mental illness, which however, are not the same for every individual. The third objective has to do with the promotion of mental health to normal persons, quite apart from any question of disease or infirmity. This is positive mental health. It consists in the protection and development of all levels of human society of secure, affectionate, and satisfying human relationships and in the reduction of hostile tensions in the community.”

There are a number of other psychiatrists who have addressed this area. One of them is Surya,[7,8] a true pioneer, who not only raised his voice against ‘colonial’ mentality, but also dared to offer alternate models for the understanding of the mind and its psychopathology. He refused to accept the ‘average or statistical norm’ as a basis for mental health. Instead, he suggested that the ‘ideal norm’ was the basis for mental health. He offered a simple three-point definition of mental health based on the Indian cultural tradition:

“Each culture provides positive and negative cues and modes of reaction leading to an integrative and creative behavior in the culture. It also provides how positively and negatively to meet threatening changes. But, in all cultures a majority of the people can only represent the negative or lower levels of behavior — so to say, a vast majority become reflex victims of their own culture. Only a minority are the reflective, conscious representatives of the best in their culture, so to say the leadership. The codes are provided by religion, philosophy, and such other structures. Moreover, some cultures are statistically oriented. That is to *say*, for them a person is in good mental health if he or she is like every other John or Jean in the neighborhood, of the same status. Economic self-sufficiency, capacity to take pleasure in occasional gossip, moderate addiction to alcohol, god and morals, is one norm. Christ and Gandhi become abnormals, fit subjects for sarcastic humor or polite patronage. There is great fear of being different from your neighbor. Other cultures are idealistically oriented. Mental Health, then, becomes an ideal goal to strive for and to achieve. Computer-calculated averages of behavior do not call for emulation. Striving toward the ideal even at the risk of being different from the neighbor becomes praiseworthy even if unsuccessful. The Indian culture pays attention to the ideal norm of its culture and striving toward that ideal is considered, by even the average person, as an important component of sound mental health. The signs of good mental health; (i) the degree to which you have an inner sense of comfort in as many situations as possible is the measure of your mental health; (ii) the faces of your intimate associates are an index of your level of mental health. The more unhappy and tense they look, or the more happy and relaxed they look in your presence is the minus and plus of your store of mental health; (iii) your account may be very poor in the above two, but the degree and duration of your aspiration and effort to change for the better is the most positive index of the state of your mental health.”

Surya also viewed common beliefs about mental health differently:

“The adjective superstition is applied to beliefs that are not current in one’s own culture.”

The fundamental, basic fact is denied, pushed away, that all normal perception is based on omission and addition of objectively non-existent data. The respectable words ‘selectivity’ of ‘perception’; ‘mental set’; ‘situational set’; anything but hallucination is used for this propose. Each culture chooses its own areas of selectivity of perception and experience, and their own criteria of reality. Greater care is needed in using such words as superstition, hallucination, etc. in reference to cultures and people as a whole”(Surya, 1966).

The importance of positive mental health is also reflected in the motto of the All India Institute of Mental Health (current NIMHANS, Bangalore), ‘Equanimity is the goal of all existence’ from the Bhagawad Gita. This approach to mental health from the early days of Independent India is an important contribution by India.[9,10]

In recent times, some of these concerns are reflected in the growing popularity of yoga and meditation as personalized approaches to better mental health, and disciplines of positive psychology.

### Classification of mental disorders

Another significant contribution of Indian Psychiatry has been in the area of classification. This is at two levels. First, is to share the Indian approach to the classification of mental disorders.[11]

“In the present state of knowledge it is not possible to have one common and universal classification, for the simple reason that we do not possess enough knowledge about the etiology of mental illness. Transcultural variations in the symptomatology of psychiatric disorders is now well known.”

The second is to influence the categories to be included in the international classification. Historically, it is remarkable that Wig and Singh[11] presented an Indian psychiatric classification, calling attention to psychiatric conditions such as hysterical psychosis. In the area of influencing international classification, the example of acute psychosis stands out as being very significant.

“Acute psychosis of uncertain etiology is one of the main innovations of our classification of psychiatric disorders.”

In ICD-8 and earlier classifications, there was no direct recognition of acute psychosis as a separate clinical entity. They were either classified as reactive psychosis or as other psychoses. It is the persistent efforts of Indian psychiatrists and the generation of research data[12,13] to support the separate clinical entity that led to the inclusion of acute psychosis as a separate category in the international classification. Furthermore, another contribution was to challenge the way some psychiatric conditions in developing countries were viewed as exotic and to move the area-specific clinical phenomenon from being viewed as a ‘native reaction’ to one of specific diagnosable conditions.[3]

### Family involvement in mental healthcare

Indian psychiatry has been a pioneer in recognizing the importance of family in mental healthcare. I consider this to be the most important contribution of Indian psychiatry to global psychiatry. At a point of time when the family of a person with mental disorder was considered ‘toxic’ in western countries, Indian psychiatrists recognied them as partners in mental healthcare. There are two aspects that have received attention, namely, the special nature of functioning of the family, and the role of the family in mental health care.

Surya[7,8] describes the Indian family and its relationship to mental health, as follows: “The important items of background in which an individual develops in the Hindu joint family, are; (i) exposure to social relationships that is spread over a number of persons — grandparents, uncles, aunts, parents, sibs, etc. The parents do not have the explicit or implicit privilege of being the sole agents for structuring social relationships and regulations for the child; (ii) as the individual grows up he or she progresses through an unending series of dependency relationships, with a large kinship circle, although with varying degrees of intensity and duration. There is no point of time at which one can look forward to a relatively free and full independent individual responsibility; (iii) marriage does not connote a landmark to the development of a fully independent unit. It marks the beginning of a new set of relationships — the recurring decimal of dependency relationships; (iv) the everlasting and ever-recurring dependency relationships are governed by concepts of inherited status. A relatively rigid status concept is divorced from the concept of role; (v) the concept of ‘mine’, ‘not mine’ is poorly developed. In an average, large joint family what rightly belongs to one and does not is never clearly demarcated.”

As a result of the paucity of organized care, families have been a part of mental healthcare throughout the history of India. Whether this was by choice or due to lack of facilities is difficult to conclude, although there is some evidence to support that family involvement in the care was, and continues to be a preference of families. The first formal recognition of the importance of the family as part of organized mental healthcare can be traced to the work of Prof. Vidyasagar in the early period of post-independent India. The next major experiment was initiated at the Mental Health Centere, C.M.C., Vellore from 1957. A very important contribution to the involvement of the family in the community care of persons with mental disorders was made by Dr. Shaila Pai, and Dr.Kapur.[14–16] Sethi[17] reflects the differing approach as follows,

“Now Indian psychiatry should also move on, to study the changing pattern of communication and interaction, so that it may fulfill the expectations of the present day population. It is widely believed that for an long time, we will not have adequate manpower of psychiatrists to handle the ever increasing number of emotionally sick individuals, hence, in planning or formulating our therapeutic intervention, we must keep the family and its role in view.”

### Psychotherapy

The subject of psychotherapy occupies an important place in the Indian psychiatry and this is one subject to which a large number of psychiatrists have contributed. Vahia[10] in his Presidential address at Srinagar observed:

“As most of the psychiatric disorders are a direct result of social and psychological stress, most of us devote some of our time in psychotherapy and yet most of us feel that we are not able to practice it as widely as we would like to. There is an urgent need to devise some methods of psychotherapy that would be useful to a large majority of our patients by the personnel in our country”.

The need for understanding the psychological origins of psychopathology has been addressed differently by Indian professionals. One of the earliest in this area was Dr. Girish Bose of Calcutta who corresponded with Sigmund Freud. Perhaps one of the most original and significant parts of this article is Dr. Surya’s observations on the concept of dependence, which is an integral part of psychoanalytical theory. Dr. Surya was the first Indian author who openly questioned the relevance of the concept of ‘dependence’ in the Indian setting.[8] After Surya, there have been others who have written more extensively on the subject,[18–23] who present an examination of the cultural relativism of dependence as a dynamic of social and therapeutic relationships[20,21] and the presentation of the Guru–Chela paradigm for psychotherapy.[19] The seeds of this line of reasoning are inherent in Surya’s earlier article of 1966.[7,8]

“In the west (UK, USA) the goal of maturity is an independent existence. There, unacceptable and unrecognized dependency longings become the focus of psychopathology and thelogy, and psychotherapy attempts to resolve these dependency needs in a manner satisfying the requirements of a culture that idealizes individual independence. In the Hindu (Indian) environment, the ideal of maturity is, satisfying the continuous dependency, striving in a manner that satisfies the requirements of a culture that idealizes individual submergence in complex interdependence. In the use of the word ‘dependency’ relationship we can already discern the language distortion and interpretation distortion I spoke of. A Western value judgment is unwittingly thrust on the people. There is no real equivalent word conveying the same value judgment. One speaks of ‘Bandha’; ‘Sambandha’; ‘Bandhvya’ — bond, bondship, kinship etc., not of dependency. It would be hazardous to import this word dependency into the Indian psychotherapeutic scene. Integration of personality functions is the Western aim. But some degree of dissociation and ideally a detachment of the higher from the lower functions is the ideal. One can speak of ‘My body is suffering. I can only watch — or I do not mind. My eyes weep but I am helpless’. The witness function of the Ego, emphasized by the Hindu thought is an important step in psychotherapy. One is encouraged to be first a nonparticipant ‘witness’ of one’s own reactions, before corrections can occur. Ours is a complex civilization. Mere cataloging of the numerous characteristics of our people from trait questionnaires drawn up in the west will give a very contradictory and distorted picture. The Kiplingisque importunate, docile, dependent untrustworthy Indian and the firm, gentle, but stern and unflinching Gandhian, Indian are two facets of the same coin.”

In the area of psychotherapy, the Bhagawad Gita, is the most widely accepted contribution of India. Dr. Venkoba Rao[24] in the book “Culture, Philosophy, Mental Health” has brought the attention of the professionals on the important value of Bhagawad Gita to mental health. Bhagawad Gita is one of the most translated religious classics in the world. The beauty and sublimity of this work, the eternal relevance to the problems of human life, and its universal approach that helps to consider the whole creation as one are the special features of this important text. The methods used by Lord Krishna to help his disciple Arjuna in the situation of war is considered by many to contain the best principles of psychotherapy. Reddy[25] relates Gita with psychoanalysis as follows. In the Gita, Krishna functions as Arjuna’s teacher and psychoanalyst. Krishna’s analytic (therapeutic) function is not interpretative per se, but more an object that facilitates the development and maturation of Arjuna’s ego (psychic). Specifically, it is Krishna’s allowing Arjuna to use him as a transformational object from a psychoanalytic viewpoint, the cardinal techniques of abstinence, anonymity, and neutrality are both observed and violated by Krishna. The pivotal and transformative violation of anonymity, by Krishna’s self-disclosure promotes the therapeutic regression and psychic reorganization that leads to Arjuna’s existential transformation. Jeste and Vahia[26] have compared the conceptualization of wisdom in ancient Indian literature with the modern views with a focus on the Bhagawad Gita.

#### Yoga and Meditation

One of Indian contributions to one’s well being in general and mental health in particular, practiced worldwide, is yoga and meditation. Information is available from the scriptures,[27,28] personal experiences,[2] and research studies.[29–33] Initial research reports on the use of yoga and meditation were with a wide range of mental disorders.[34–36] This was followed by comparison of standard treatment with yoga in psychoneuroses, anxiety, drug addiction, and psychogenic headache. There were also a number of studies on the various aspects of Transcendental Meditation and its physiological effects. The more recent studies have examined the effectiveness of specific treatments based on Sudarshan Kriya Yoga (SKY) in dysthymia, depression, schizophrenia, and drug and alcohol dependence. The increased interest in eastern therapies and the availability of measures to study the effects should result in more sophisticated studies on the effectiveness of the different therapies in different mental disorders. There is also a re-examination of ancient Indian wisdom to modern mental health practice.[26,37,38]

### Understanding of mental disorders

Indian psychiatrists of the 1950s,’60s, and ’70s, more than any other generation of psychiatrists, were aware of the gap between the training they had received and the day-to-day practice of psychiatry in India. As a consequence, one of the running themes in the professional lives of this group of psychiatrists was the need to understand the nature of mental disorders in India.[3] On the professional front, almost all the group members addressed this theme in various forms — ranging from epidemiological studies, descriptive studies, systematic analysis of clinical records, specific studies of course, and the outcome of mental disorders, to share the Indian understanding of mental disorders.

One of the important and early pioneers in this effort was Dr. Govindaswamy.[6] He was the first to think of a broad-based general population psychiatric epidemiological study, in Bangalore city. Around 1957, he viewed the importance as follows:

“We ought to begin our discussion of the progress of psychiatry in India by considering the incidence of mental disorders in the country. But unfortunately, no reliable statistics bearing on this matter are available. Neither the admission rates not the number of beds occupied in mental hospitals can be considered reliable indices of the actual number of patients suffering from mental disorders in the area served by these hospitals for the following reasons….”

Although Dr. Govindaswamy made a big effort to launch an epidemiological study, the very ambitious goals to ‘find the causes’ contributed to its non-fruition. It was only a decade later that others took up this activity and completed some of the pioneering epidemiological studies. The first of this was by Dr. Surya, in 1964, to complete the first general population epidemiological study. This was soon followed by the historic mile stone of the Agra epidemiological study lead by Dr. Dube. The importance of the Agra study by Dr. Dube[38] can be gauged by the fact that till today, it continues to be the most important contribution in this field. Soon, other leaders took up the study in other parts of the country. Dr. Sethi’s study of 300 urban families,[39] 500 rural families,[40] and the prevalence of mental retardation in Lucknow[41] is an important contribution. Other centers that took the lead further were the Vellore group,[42] the Calcutta group led by Dr. Nandi.[43] The Great Universe of Kota[44] was an important milestone in Indian psychiatric epidemiology, which impacted western psychiatry. The impact of the efforts of these pioneers was the recognition of the public importance of mental disorders in the country.[45]

Along with the understanding of the magnitude of mental disorders, there were efforts to describe the symptoms of different mental disorders, as seen in Indian patients. I recall in my early days of psychiatric work in the 1970s, the debates about the North–South differences in the prevalence and pattern of depression. Simultaneously, Dr. Vahia, Dr. Bagadia, Dr. Sethi, Dr. Hoch, Dr. Bhaskaran described the differing ways mental disorders presented in the Indian population. The role of culture in depression was described by Venkoba Rao,[46] following a study of a group of patients with depression as follows:

“Considering all these factors, ideas of guilt (both karmic and non-karmic) do not appear to be integral to the depressive illness in India. Nevertheless, further work is clearly called for, to support or contradict this observation”.

This series of studies to understand the course and outcome was very important as they provided new insights such as better prognosis in schizophrenia in India. The contribution of Prof. Dube as part of the WHO International study on schizophrenia during the 1960s and 1970s,[47] and the ICMR, New Delhi supported the study ‘Study of factors associated with the course and outcome of schizophrenia (SOFACOS)’[13,48–50] and the focused and longitudinal studies from Chennai and Chandigarh have kept the debate of the reasons for the differing course of schizophrenia a live topic.

### Community care of mentally ill persons

This is an area of concern among Indian psychiatrists during the last 60 years, as they were acutely aware of the wide gap between the needs and the resources available.

Vahia[51] addressed the issue of services, in his Presidential address at Srinagar in 1966, as follows;

“Considering the lack of availability of trained personnel we will have to plan to make maximum use of the existing personnel. For this purpose, the psychiatrists from the mental hospitals will have to come out of the hospitals and provide out-patient treatment facilities. Even that is not enough, as many mental hospitals are away from the cities and towns, and as it is difficult for the patients to go there, the psychiatrists will have to come out of their ivory tower and work in general hospitals by developing out-patient and in-patient treatment facilities for children and adults. When other countries are planning to develop community mental centers so that the patient can live and work in the community during and soon after treatment, we should plan to reach out into the community and make ourselves readily available to our patients, so that they can be helped as soon as possible”.

Bhaskaran[52,53] in his 1971 Presidential address of the Indian Psychiatric Society, at Hyderabad, addressed the issue of mental health services extensively under the title ‘unwanted patient’. He called for a number of interventions to address the needs of patients.

“There seems to be a general consensus that traditional large mental hospitals have outlived their usefulness and may actually serve as breeding grounds for secondary problems like hospitalism, in addition to continuing to serve the purpose of a dumping ground for incarcerating the unwanted chronic mentally ill. Such institutions should ever be built in future (emphasis added). There is enough convincing evidence to show that schizophrenic patients of all types and stages of illness can be successfully treated in smaller, open institutions situated more centrally in the community. To really convince the average man of the treatability and returnability of the mentally ill person to the community as a useful citizen, one must show concrete results and we have not even made an effort in this direction in our country, and this only underscores the need for immediate and energetic rehabilitation of the more chronic patients. There is a need for the launching of pilot projects in the home care of schizophrenic patients, training a cadre of social workers and psychiatric nurses for the aftercare of discharged patients, and launching pilot projects engaging college students as volunteers in the resocialization of chronic schizophrenics”.

Prof. D. Satyanand started one of the first studies on community psychiatry where a team from AIIMS, New Delhi, was going regularly to a rural center in Ballabhgarth.

Sethi[54] in an Indian Journal of Psychiatry, editorial called for action in this area as follows:

“We must strongly suggest to the planners, thinkers, and implimentators to accord a super-speed priority in evolving the strategies of tomorrow and to ensure a very early adoption of a National Mental Health Program, now under active consideration of the National and state authorities”.

A visible outcome of the efforts of professionals like Bhaskaran,[52,53] Sethi,[54] Wig[55] and Kapur,[56,57] and others, is the formulation of the National Mental Health Program (NMHP), in 1982.[58] NMHP provided both the direction for the organization of services and the models for reaching there. The central idea was described by the professionals as an integration of mental health with general health services. This initiative, initially started at the Chandigarh and Bangalore centers,[56,57,59] has resulted in a significant difference in mental health planning in India during the last three decades. From the initial studies of the 1970s involving small population units of about 50,000, today it covers over 125 districts in the country.[58] In addition, the experiences of the Indian approach to community mental healthcare, through integration of mental health with general health services, have directly influenced the development of mental health services in Afghanistan, Bangladesh, Bhutan, Islamic Republic of Iran, Nepal, Pakistan, Palestine, Srilanka, Sudan, and Yemen through professional support in the form of consultancy support, training opportunities, and research.[60] Moreover, another aspect of community care is the large number of community care facilities initiated by the voluntary organisation.[61]

### Traditional systems of mental health care

In India a highly developed and elaborate system of medicine has flourished for nearly three thousand years.[27,28,37] It is generally known by the name of Ayurveda (the science of life). There are many medical texts dating back to the first and second century AD, which describe in detail the principles of Ayurveda. The two best known medical works are by the Ayurvedic physicians Charaka and Sushrutha. These books were originally compiled sometime between the third century BC and third century A.D. The principles of Ayurvedic medicine, like in other Indian philosophical systems, were probably well developed by third century BC.

Dube[62,63] has systematically examined the nosology and therapy of mental illness in Ayurveda by comparing the clinical conditions described in Ayurveda with the clinical conditions described in the International Classification of Diseases. Dube *et al*.,[64] describe the selection and training procedures, ethical codes, metaphysical theories, and principles of psychiatric treatments as practiced in Ayurveda.

“Three treatment approaches for psychological disorders are: ministered therapies (psychotherapies), performance therapies (rituals), auto-therapies. Other forms of therapies mentioned are herbal, physical, and natural. Treatments were administered with benediction. Man was treated as a whole with a psychosomatic approach. The therapeutic measures for insanities varied from words of sympathy and comfort to terrorizing by means of snakes, from purgation to venesection. The other measures were purification procedures by emetics and diaphoretics. If they were of no avail, ocular and nasal instillations with medicated ghee were recommended. The drugs used included colosynth, pepper, valerian, turmeric, indian sarpasarilla, cardamom, cinnamon, leaf, sandalwood, garlic, pomegranate, jejube, radish, ginger, and asafetida, goat’s and cow’s urine, and of ox and jackal bile were used as vehicles. In some instances of disoriented mind such measures as anointing with mustard oil, exposure to sunlight, branding with hot irons, or scourging with a whip were recommended. Terrorizing by snakes whose fangs had been removed or by trained elephants or lions, or by men dressed as bandits or men with weapons, and intimidation with threats of immediate execution were employed when all other measures had failed on the plea that threat of life is more patent than fear of bodily injury. Rowalfia serpentina was a popular drug for insanity in ancient India. Known as Sarpagandha in Sanskrit, it was used in treating a variety of diseases and symptoms ranging from constipation to insanity”.

## CONCLUSION

Reviewing the Indian contributions to global psychiatry, it is impressive to see the constant efforts of Indian psychiatrists to relate and contribute to world psychiatry. However, two aspects stand out as needs for the future. First, there is a need for greater focused and in-depth examination of the areas identified and worked upon by psychiatrists.[65] Second, there is a greater need for documentation and critical analysis of the contributions.

As noted in the beginning of the article, Indian psychiatry has interacted with global psychiatry by both contributing and benefiting from world psychiatry. This two-way process will be an ongoing activity in the coming years. Reviewing the developments, two other thoughts are worth reflecting on.

There is need for Indian psychiatry to go beyond sitting on the richness of ancient wisdom. This was noted by Prof. Venkoba Rao.[66]

“India is an ancient and great cultural, spiritual, and anthropological laboratory. She has been the nursery of saints and sages, scientists and founders of the world’s major religions and promulgators of profound philosophy. Nevertheless, to be satisfied with the glory of the past is to turn into a fossil; but to interpret the old from a new point of view is to revitalize the past and bring in a current of fresh air into the monotonous present.” This is the challenge and opportunity for the mental health professionals of India and the world.

Another aspect calling for greater effort from Indian psychiatrists, toward influencing world psychiatry, is the need for greater research into the Indian concepts and practices. This was noted by Carstairs.[67]

“One has to admit that there is little firm evidence that either meditation or religious observance significantly modifies, tens of thousands of Indians, young and old, have become disciples of teachers who support them in their twofold ambition to practice right conduct in accordance with Hindu dharma and to enhance their personalities by following a particular technique of meditation. If it could be established, with appropriate control, that changes in symptoms and in personality traits do come about, and in the desired direction, then the possibility of collaborating between psychiatrists and Gurus could be worth exploring”.

The contributions of Indian psychiatry to world psychiatry, reviewed in this article, in the fields of family involvement in mental healthcare, classification, study of course, and outcome of mental disorders, yoga and meditation, and community care of mental disorders, illustrates the way Indian psychiatry can continue to contribute and influence world psychiatry in future.
